# Eyelash trichomegaly and durable complete response in a patient with metastatic lung adenocarcinoma receiving tyrosine kinase inhibitors: a case report

**DOI:** 10.1186/s13256-026-06170-0

**Published:** 2026-06-05

**Authors:** Afraa Baghdad, Kloda Ali, Mousa Alali, Maher Saifo

**Affiliations:** 1 Faculty of Medicine, Damascus University, Fayez Mansour Street, P. O. Box: 222 Damascus, Syrian Arab Republic; 2 Department of Oncology, Faculty of Medicine, Albairouni University Hospital, Damascus University, Harasta M5, Damascus, Syrian Arab Republic

**Keywords:** Epidermal growth factor receptor, Erlotinib, Gefitinib, Eyelashes, Non-small cell lung cancer, Case report

## Abstract

**Background:**

Erlotinib and gefitinib are oral first-generation tyrosine kinase inhibitors (TKIs) approved for the treatment of advanced non-small cell lung cancer (NSCLC) harboring epidermal growth factor receptor (*EGFR*) mutations. The most common adverse effects of these TKIs include rash and diarrhea. Eyelash trichomegaly is a rare adverse effect and not considered drug-limiting. This report presents a case of long-term complete response and TKI-induced eyelash trichomegaly in a patient with metastatic lung adenocarcinoma.

**Case presentation:**

A 47-year-old Syrian woman with *EGFR*-positive recurrent metastatic lung adenocarcinoma was treated with gefitinib or erlotinib (as available) as monotherapy for 42 months and a complete response was achieved. After initiating erlotinib, the patient developed eyelash trichomegaly, which was managed conservatively with occasional trimming. When erlotinib treatment was discontinued, the elongated eyelashes regressed.

**Conclusions:**

This case demonstrates the effectiveness of first-generation TKIs in treating metastatic *EGFR*-positive NSCLC, particularly in countries that cannot afford recent targeted therapies. In addition, it describes a rare adverse effect that was well tolerated and managed successfully.

## Introduction

Lung cancer is the most common cancer and the leading cause of cancer-related death worldwide [1]. The two major histologic types of lung cancer are non-small cell lung cancer (NSCLC), accounting for 80–85%, and small cell lung cancer, accounting for 15–20%. Adenocarcinoma is the most common histologic subtype of NSCLC [2, 3].

Epidermal growth factor receptor (EGFR) is a tyrosine kinase receptor. When its ligands bind to cell-surface receptors, they trigger receptor dimerization, tyrosine kinase activation, and autophosphorylation, initiating intracellular signaling cascades. Somatic *EGFR* mutations in NSCLC may result in constitutive activation of downstream signaling pathways [4]. *EGFR* mutations are observed in approximately 10% to 30% of adenocarcinomas. Approximately 90% of *EGFR* mutations are exon 19 deletion and point mutation in exon 21 (L858R), both of which confer sensitivity to EGFR tyrosine kinase inhibitors (TKIs), such as erlotinib, gefitinib, and osimertinib [5, 6].

One of the most common side effects of TKIs is cutaneous toxicity [7, 8]. These reactions, including acneiform rash and trichomegaly, occur because EGFR is expressed in keratinocytes, the suprabasal layers of the epidermis, and the outer root sheath of hair follicles [9]. Erlotinib and gefitinib (first-generation TKIs) are oral agents that used in the treatment of advanced and metastatic NSCLC. This report presents a rare case of a woman with metastatic lung adenocarcinoma treated with erlotinib and gefitinib, who developed elongated eyelashes, along with a durable complete response.

## Case presentation

A 47-year-old Syrian woman, married with three children, presented to our cancer center in 2021 with dyspnea and cough due to a relapse of previously treated NSCLC. She is a nonsmoker and does not consume alcohol. She has a history of hypothyroidism, for which she is receiving thyroxine. Her family history is notable for hypothyroidism in her mother and sister, both treated with thyroxine. In addition, her mother has non-small cell lung cancer, her sister has invasive ductal carcinoma of breast, and her brother is currently being treated with TKIs for metastatic renal cell carcinoma.

In March 2020, the patient was diagnosed with a stage IIIA adenocarcinoma of the left lung. She underwent a complete pneumonectomy, followed by six cycles of adjuvant chemotherapy (carboplatin/vinorelbine) and subsequently received adjuvant radiotherapy, with follow-up every 3 months.

In January 2021, the patient complained of dyspnea and cough. Positron emission tomography computed tomography (PET-CT) revealed numerous irregulars and scattered densities within the right lung, with metabolic activity (SUV 2–5) consistent with recurrence. A new CT-guided biopsy was obtained, and pathological evaluation confirmed adenocarcinoma. Molecular testing via real-time PCR identified an *EGFR* mutation (exon 19 deletion, delE746-A750). Based on these findings, treatment with gefitinib 250 mg daily was initiated in February 2021. After 3 months of therapy, a CT scan demonstrated a complete response. The plan was to continue gefitinib and perform CT or PET-CT every 3–6 months for evaluation.

During the treatment, the patient reported mild facial and neck rash, conjunctivitis, dry skin, loss of appetite, and mild diarrhea. Laboratory tests, including complete blood count, renal function tests, thyroid-stimulating hormone, and liver function tests were within the normal limits.

**Fig. 1:**
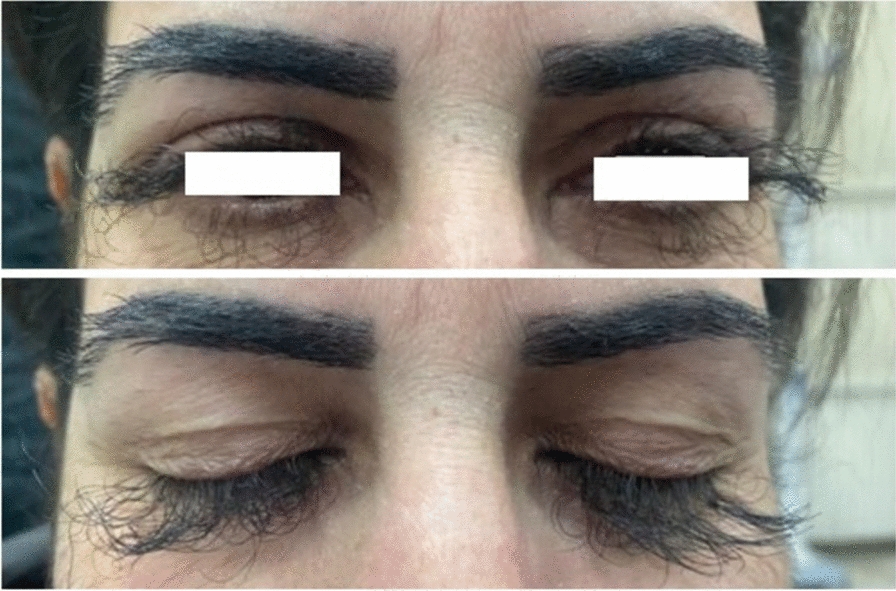
Eyelash trichomegaly in a patient with metastatic lung adenocarcinoma receiving tyrosine kinase inhibitors

In March 2023, the patient was switched to erlotinib 150 mg once daily for 9 months due to the unavailability of gefitinib at the hospital. One month after initiating erlotinib, she developed notable elongation of the eyelashes on both eyelids, while continuing to experience the same skin-related symptoms (Fig. 1). The eyelashes growth was considerable, necessitating repeated trimming with scissors. No changes in vision or ocular symptoms were observed, and no additional complaints were reported. Table 1 presents a comparison of erlotinib and gefitinib with respect to side effects and efficacy in this patient.

**Table 1:** Side-by-side comparison of erlotinib and gefitinib in terms of side effects and efficacy

Adverse effect	Erlotinib	Gefitinib
Eyelash trichomegaly	Yes	No
Skin rash	Yes	Yes
Diarrhea	No	Yes
Conjunctivitis	No	Yes
Loss of appetite	Yes	Yes
Hematologic effects	No	No
Hepatotoxicity	No	No
efficacy	Complete response	Complete response

**Fig. 2:**
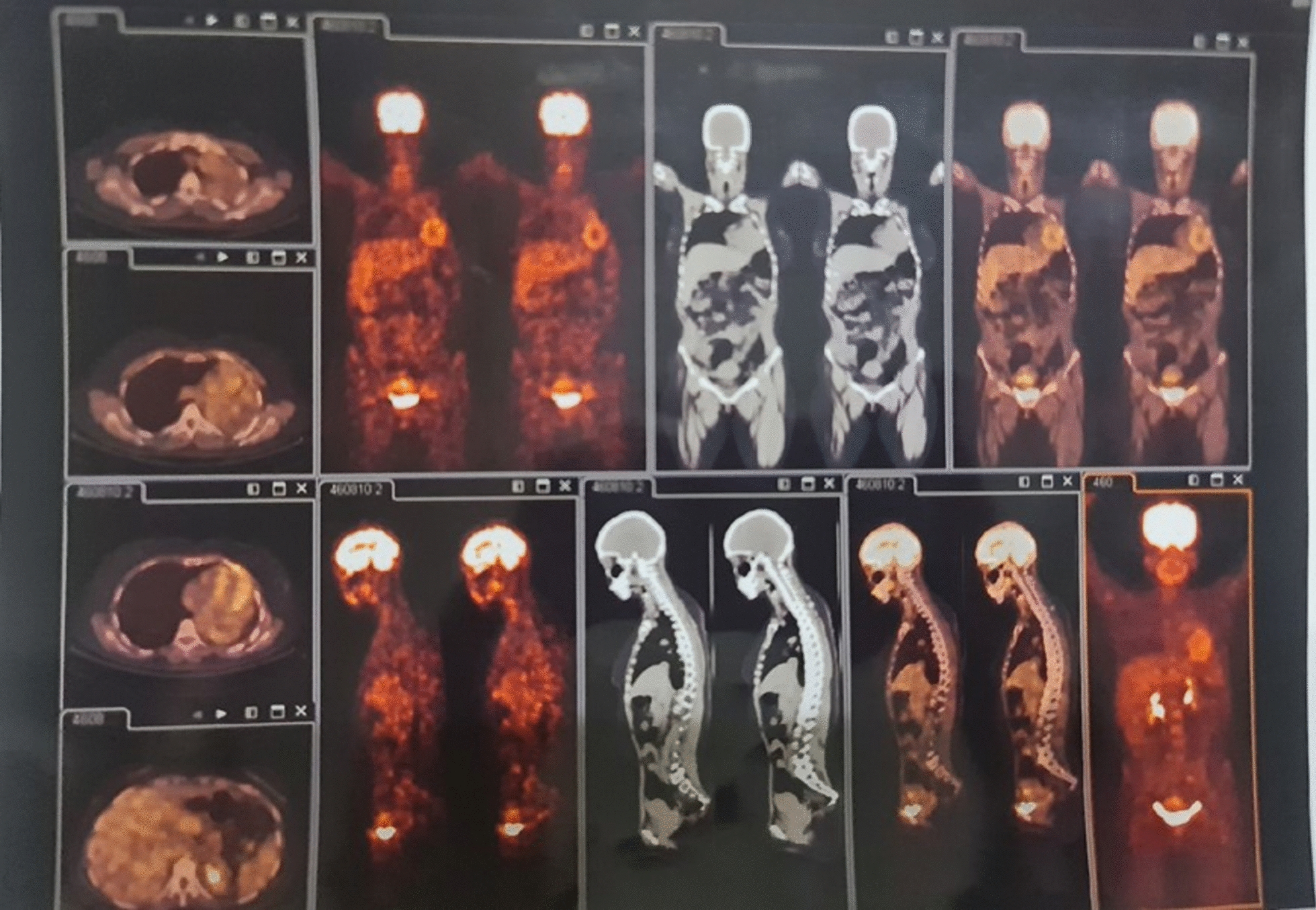
A positron emission tomography–computed tomography showed a complete response to tyrosine kinase inhibitors in a patient with recurrent lung adenocarcinoma. The scan shows left lung resected with fibrosis in its place

**Fig. 3:**
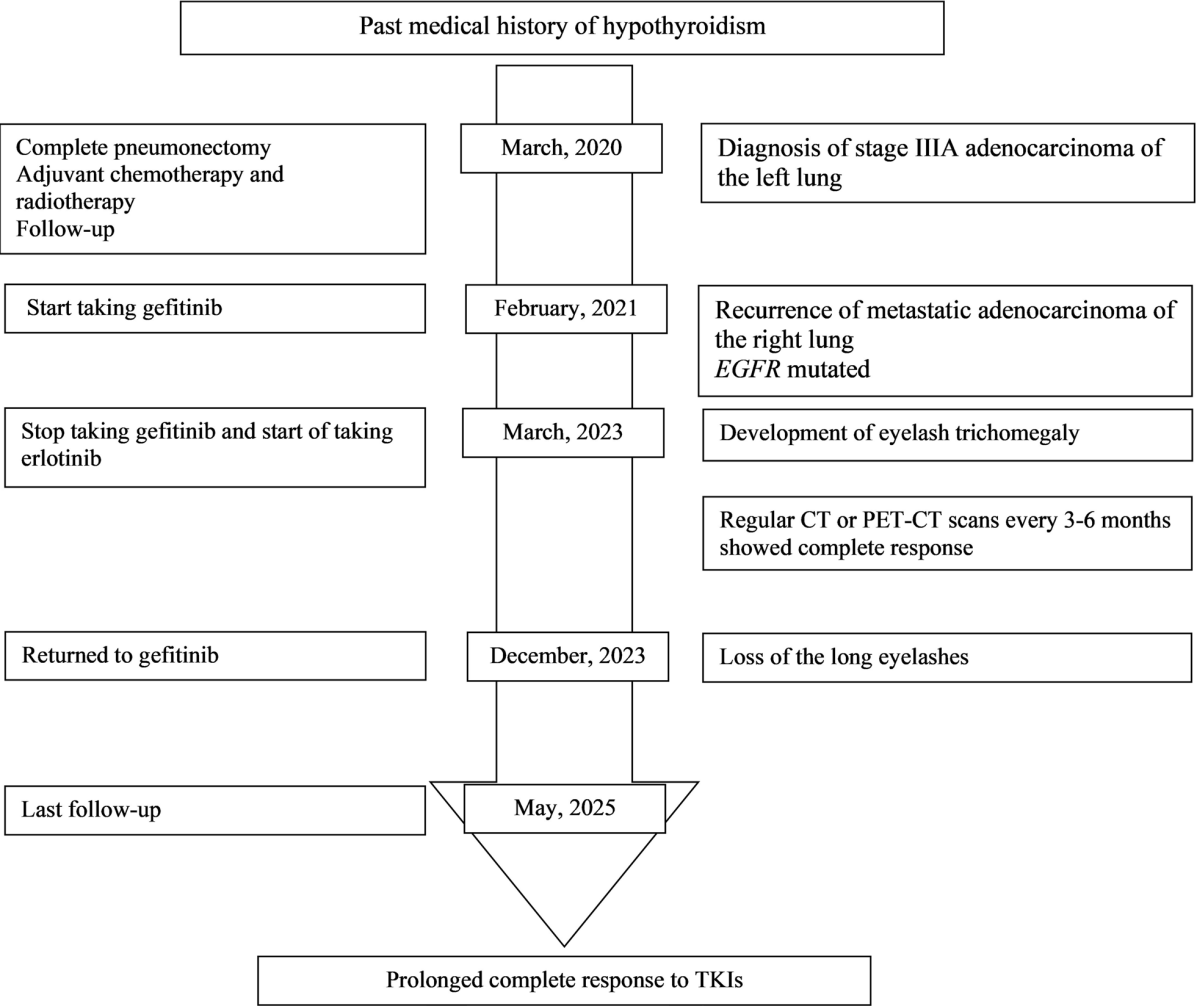
The timeline for the patient’s case. *CT *Computed tomography, *EGFR* Epidermal growth factor receptor, *PET* Positron emission tomography, *TKIs* tyrosine kinase inhibitors

Following the cessation of erlotinib and the resumption of gefitinib therapy, the patient experienced loss of her eyelashes. The three most recent imaging evaluations have confirmed a complete response, and nearly 4 years after relapse, she remains free of tumor progression and in good health (Fig. 2). The timeline for the patient’s case is illustrated in Fig. 3. This case was previously reported in 2022 before initiation of erlotinib therapy [10]. The current report describes new clinical findings, including eyelash trichomegaly following erlotinib treatment.

## Discussion and conclusions

In this report, we present a rare case of a patient with relapsed NSCLC who achieved a complete response to TKI therapy and developed a rare side effect of eyelash lengthening. To our knowledge, this represents the first documented case in Syria.

EGFR is expressed in both basal keratinocytes and external root-sheath cells, where it plays a role in regulating the phases of the hair growth cycle, particularly the transition between anagen and catagen phases. At the molecular level, EGFR signaling activates downstream pathways such as the RAS–RAF–MAPK and PI3K–AKT cascades, which promote keratinocyte proliferation, survival, and controlled differentiation. In the hair follicle, EGFR signaling also modulates stem cell activity within the bulge region and coordinates interactions between follicular keratinocytes and the surrounding dermal microenvironment. Pharmacologic inhibition of EGFR disrupts these pathways, leading to impaired keratinocyte proliferation and premature differentiation, along with increased apoptosis. In hair follicles, this results in dysregulation of the normal hair cycle, often promoting premature entry into catagen and impairing proper anagen maintenance. Additionally, EGFR inhibition alters cytokine and chemokine expression (including upregulation of IL-1, IL-6, and TNF-α), contributing to a pro-inflammatory milieu within the follicular unit. These side effects collectively known as PRIDE syndrome, include papulopustules and/or paronychia, regulatory abnormalities of hair growth, itching, and dryness, these manifestations reflect both direct effects on epidermal homeostasis and secondary inflammatory responses. In the context of hair follicles, EGFR blockade has been associated with alterations in hair shaft formation and cycling dynamics, which may contribute to clinical findings such as trichomegaly, as described in previous reports [11, 12].

Eyelash trichomegaly is a rare side effect characterized by an excessive increase in the length, thickness, stiffness, curling, or pigmentation of the eyelashes. It is associated with the use of certain drugs, including EGFR inhibitors such as cetuximab, gefitinib, erlotinib, and panitumumab [13]. Despite the widespread clinical use of erlotinib in metastatic NSCLC, the prevalence of trichomegaly in these patients remains poorly defined [14].

In our case, the patient received erlotinib and gefitinib and experienced dermatologic toxicity with both agents. Notably, erlotinib induced eyelash trichomegaly, whereas gefitinib did not. Following discontinuation of erlotinib and resumption of gefitinib, the patient experienced loss of the elongated eyelashes, and this suggesting that erlotinib may be associated with eyelash elongation. However, this association is observational and does not establish a definitive causal relationship. Trimming the eyelashes with scissors proved to be a practical management option for this side effect, as demonstrated in this case [13].

It is noteworthy that not all side effects are clinically undesirable; some may be therapeutically beneficial or intentionally utilized in specific contexts. For example, the antihistamine cyproheptadine is commonly prescribed to stimulate appetite in patients with cancer [15]. In the present case, the patient experienced eyelash elongation (trichomegaly), which was well tolerated and did not result in reported discomfort.

Owing to their distinct chemical structures and differing receptor-binding affinities and toxicity profiles, gefitinib and erlotinib, despite demonstrating comparable efficacy, may be considered partially interchangeable in the treatment of patients with advanced NSCLC harboring *EGFR* mutations. However, given the molecular and pharmacokinetic differences between these agents, their safety profiles are not identical. Gefitinib is more frequently associated with gastrointestinal adverse effects, whereas erlotinib tends to be associated with a higher incidence of dermatologic toxicities [16].

Several studies have suggested that the development of cutaneous toxicities is associated with a more favorable response and improved survival outcomes in patients treated with erlotinib. However, the clinical significance of trichomegaly remains unclear. To date, several published studies, along with the current report, have indicated that tumor response may be observed in patients who develop trichomegaly. However, most previous studies are case reports or retrospective studies; therefore, prospective studies with larger sample sizes are required to determine whether trichomegaly or cutaneous toxicities can serve as predictive factors [14]. Table 2 provides a summary of reported cases of eyelash trichomegaly following erlotinib treatment [17–30].

**Table 2:** Reported cases of eyelash trichomegaly after erlotinib

Year/author	Age (years)	Study	Sex	Stage	Tumor, histology	Management of trichomegaly
This case	47	Case report	Female	4	Lung, adenocarcinoma	Eyelash trimmings
Celik T [17]	74	Case report	Female	4	Lung, adenocarcinoma	
Jeon SH [18]	52	Case report	Female	4	Lung, adenocarcinoma	Eyelash trimmings
Zheng H [19]	29, 85, 68, 75, 77, 60	Case series (6 cases)	2 Females, 4 males	4	Lung, adenocarcinoma	NS
Lane K [20]	70	Case report	Female	NS	Lung, adenocarcinoma	Eyelash trimming
Wang SB [21]	65	Case report	Female	4	Lung, adenocarcinoma	Eyelash trimming
Papadopoulos R [26]	72	Case report	Female	4	Lung, adenocarcinoma	Epilation or treated with electrolysis
Saif MW [22]	75	Case report	Male	4	Pancreas, NS	NS
El Haddad M [30]	50	Case report	Female	NS	Lung, adenocarcinoma	Eyelash trimming
Goel V [27]	39	Case report	Male	4	Pancreas, adenocarcinoma	Eyelash trimming
Kremer N [23]	58, 72, 79	Case series	Females	NS	Lung, adenocarcinoma	
Kau HC [28]	63	Case report	Male	4	Lung, adenocarcinoma	Eye drop treatment
Desai RU [29]	70	Case report	Male	Advanced stage	Lung, adenocarcinoma	Eyelash trimming
Munoz J [24]	48	Case report	Females	Advanced stage	Lung, adenocarcinoma	Eyelash trimming
Vergou T [25]	74, 60	Case series	Females	4, advanced stage	Lung, adenocarcinoma	Hair removal

The study by Soria *et al*. [31] compared osimertinib with first-generation TKIs (gefitinib and erlotinib) and demonstrated that a progression-free survival (PFS) was 18.9 months versus 10.2 months, respectively. In the present report, a durable response to first-generation TKIs was observed, with PFS of 42 months until now. In our study, the use of osimertinib as a first-line treatment for recurrent disease was not feasible due to its unavailability at the hospital at the time of diagnosis [32]. However, owing to limited resources and frequent drug shortages, it was necessary to alternate between gefitinib and erlotinib during the course of treatment. Furthermore, second-generation EGFR-TKIs, as well as ALK, ROS1, and TRK inhibitors, in addition to immune checkpoint inhibitors, remain largely inaccessible in many low-income countries due to their high cost [3].

Given the patient’s family history of cancer, a hereditary cancer predisposition could not be excluded; however, genetic testing was not performed due to lack of insurance coverage and financial constraints.

In conclusion, this report describes a rare case of a patient with relapsed NSCLC who achieved a complete response to TKI therapy with gefitinib and erlotinib. Notably, the patient experienced a rare side effect characterized by eyelash elongation (trichomegaly), which appeared to be associated with erlotinib treatment. This case also highlights the potential utility of first-generation TKIs in recurrent *EGFR*-mutant NSCLC, particularly in resource-limited settings where access to newer targeted therapies or comprehensive molecular testing may be restricted.

## Disclosure

This is an original manuscript that has not been previously published and is not being submitted elsewhere.

## Data Availability

The data supporting the findings of this study are available from the corresponding author upon reasonable request**.**

## Abbreviations

CT: Computed tomography
EGFR: Epidermal growth factor receptor
NSCLC: Non-small cell lung cancer
PET: Positron emission tomography
TKIs: Tyrosine kinase inhibitors
